# Emotional framing in online environmental activism: Pairing a Twitter study with an offline experiment

**DOI:** 10.3389/fpsyg.2022.1099331

**Published:** 2023-01-30

**Authors:** Mary Sanford, Marta Witkowska, Robert Gifford, Magda Formanowicz

**Affiliations:** ^1^Oxford Internet Institute, University of Oxford, Oxford, United Kingdom; ^2^Environmental, Social and Personality Lab, Department of Psychology, University of Social Sciences and Humanities, Warsaw, Poland; ^3^Center for Research on Social Relations, University of Victoria, Victoria, BC, Canada

**Keywords:** climate change, activism, Twitter, emotion, framing, mood, suppression, psychology

## Abstract

As the consequences of anthropogenic climate change become more apparent, social media has become a central tool for environmental activists to raise awareness and to mobilize society. In two studies, we examine how the emotional framing of messages posted by environmental activists influences engagement and behavioral intentions toward environmental action. In the first study, tweets (*N* = 510k) of 50 environmental activists posted between November 2015 and December 2020 are examined to measure their emotional content and its relation to tweet diffusion. Environment-related tweets are found to be shared more the less they contain positive emotion and the more they contain negative emotion. This result supports the negativity bias on social media. In Study 2 (*N* = 200), we experimentally test whether negatively vs. positively framed environmental content leads to increased reported intent to engage with collective action, and whether mood mediates that link. We find both direct and indirect effects on reported climate action intentions when mood is used as a mediator. The negative mood resulting from seeing negative tweets makes participants more likely to report higher action intention (indirect effect)—congruent with Study 1. However, seeing negative tweets also makes participants less inclined to act (direct effect), indicating a suppression effect and the presence of other factors at work on the pathway between information and action intent formation. This work highlights the complex and multifaceted nature of this relation and motivates more experimental work to identify other relevant factors, as well as how they relate to one another.

## 1. Introduction

Social media platforms have become a critical venue for environmental activism and communication. These platforms have opened up direct communication and conversation channels between the general public and users seeking to raise awareness and mobilize this public toward climate action (Pearce et al., 2015). Such users, referred to in this paper generally as activists, include climate scientists and journalists, Green politicians, and celebrities, who use their influence stature to communicate with the public about environmental issues.

These environmental activists can be seen as “opinion leaders,” originally conceptualized by Lazarsfeld et al. (1968) as knowledgeable, respected, and socially-active mediators of public opinion. Previous work in environmental communication demonstrates how opinion leaders shape the social media discourses in which they operate and have strong mobilizing power for pro-environmental action (Han and Ahn, 2020; Boatwright, 2022; Dekoninck and Schmuck, 2022). However, most research examining environmental communication on social media has taken a more general approach, by tracking all content linked to popular terms or hashtags, in order to capture the aggregate tenor and topical distribution of a given discourse context. These analyzes treat users and their posts as “sensors” emitting signals, which when pooled together can provide an indication about public understanding and opinion on different topics (Boyd and Crawford, 2012; Kirilenko and Stepchenkova, 2014; Kirilenko et al., 2015).

This kind of analysis helps to summarize various characteristics of a discourse, but not to better understand how prominent individuals or organizations may be shaping it. While some studies identify the most prominent or influential users and/or narratives in a discourse (Kirilenko and Stepchenkova, 2014; Pearce et al., 2014; Williams et al., 2015; Newman, 2017; Mavrodieva et al., 2019; Chen et al., 2022), few have focused on opinion leaders and the strategies they use to try to capture the public's attention and persuade them to act. Therefore, by examining activists specifically we deepen the field's understanding of how key communicators may be impacting the development of environmental discourses, as well as what can be done to optimize this trajectory toward maximum awareness and mobilization.

Meanwhile, experimental work in environmental psychology demonstrates that the emotional frames that communicators embed in messages about environmental issues differentially impacts an audience's reaction to and engagement with this information. Yet, none of this work has focused on emotional framing in environmental communication disseminated on social media platforms, nor on the messaging of activists operating on them in order to harness their potential for awareness building, persuasion, and mobilization. Instead, researchers have focused predominantly on measuring the overall sentiment of a given discourse context, or in offline studies, testing the effects of emotional framing in longer form text, e.g., newspaper articles. Given the rising prominence of social media platforms as a source of news and general information (Newman, 2021; Shearer and Mitchell, 2021; Walker and Matsa, 2021), as well as the declining rates of users consuming full-length articles (Haile, 2014; Thurman, 2014; Dunaway et al., 2018; Martin, 2018), it is important to consider engagement with emotional framing in social media contexts.

The present work seeks to contribute to this research area with two studies. First, we investigate whether different emotional framing in the posts of environmental activists on Twitter sparks correspondingly differential engagement from their audiences in the form of retweets. In the second study, emotional framing in tweets is experimentally manipulated to see whether results obtained in Study 1 can be replicated in an offline setting, and to investigate a pathway potentially affecting reported behavioral intentions. Before we introduce the studies, we detail the empirical and theoretical context of the work.

## 2. Literature review

Social media platforms have become an important arena for environmental communication, activism, and mobilization (Schäfer and Schlichting, 2014; Koteyko et al., 2015; Anderson, 2017; Lörcher and Taddicken, 2017; Mavrodieva et al., 2019; Boulianne et al., 2020). An extensive body of literature has accumulated in recent years on how environmental issues have been presented and discussed on social media platforms. A major finding of this work has been the extent to which environmental discourses tend toward controversy (Wiest et al., 2015; Olausson, 2018; Sanford et al., 2021), negativity (Dahal et al., 2019; Loureiro and Alló, 2020; Tyagi et al., 2020; Sanford et al., 2021), and polarization (Jang and Hart, 2015; Williams et al., 2015; Garcia et al., 2019; Falkenberg et al., 2022). However, very few studies have examined the role of specific thought or opinion leaders in influencing these trends. New research shows that individuals who follow environmental activists on social media have stronger pro-environmental convictions and behavioral intentions (Dekoninck and Schmuck, 2022). Yet, we have little understanding of how the content of the messages shared by activists relates to public engagement with activist information campaigns, both on and offline.

One component that previous experimental work with other forms of environmental communication, e.g., news articles, government leaflets, and videos, has identified as salient for shaping pro-environmental beliefs and behavior is emotion. Work in this literature typically compares the relative impact of framing environmental communication stimuli with positive emotions, such as hope and empowerment, to the impact of framing the stimuli with negative emotions, such as fear, worry, and anger, on a variety of pro-environmental behavioral and attitudinal outcomes.

One branch of work in this area finds a clear positive relationship between information containing positive emotions and participants' attention and pro-environmental behavioral intentions (Ojala, 2012; Chadwick, 2014; Feldman and Hart, 2016; Nabi et al., 2018; Wang et al., 2022). Further research reports a complementary detrimental impact of negative emotion in environmental communication. Generally, communication that contains negative messages with a focus on risk, threat, and danger tend to trigger emotional responses leading to feelings of helplessness, apathy, and contempt, all of which decrease attention and behavioral intention (Loewenstein et al., 2001; Lowe, 2006). In a study of the responses of adolescents in Australia to negatively-framed climate change education, the authors found the majority report feeling disempowered and helpless regarding climate change (Jones and Davison, 2021). Similarly, Feinberg and Willer (2011) find that when exposed to fear-mongering about “apocalyptic consequences” of climate change in newspaper articles and videos, participants report higher levels of apathy, denial, and avoidance instead of action.

These studies suggest that negative emotions make individuals feel less hopeful about the possibility to combat climate change, and therefore less likely to partake in climate action, while usage of positive emotions can be motivational and engaging. However, another body of scholarship suggests the opposite relation. Several experimental researchers have found that negative emotions can help focus attention on environmental issues and motivate deeper considerations of possible solutions than information framed with positive sentiment (Meijnders et al., 2001a,b; Hine et al., 2016; Bouman et al., 2020; DiRusso and Myrick, 2021).

Moreover, based on the work of Baumeister et al. (2001) and Rozin and Royzman (2001), we know that individuals have a tendency to pay more attention to negative (bad news) than to positive content (good news) in media communication. As a consequence, it is possible that this negativity bias has precipitated an emphasis on negative content in environmental communication on social media and in mainstream news media. Indeed, several studies of coverage and communication of environmental issues in both arenas confirm a general trend toward negative sentiment (Wiest et al., 2015; Dahal et al., 2019; Loureiro and Alló, 2020; Tyagi et al., 2020; Sanford et al., 2021).

Not only is negative sentiment more prevalent, but it may also be more engaging for some audiences. Del Vicario et al. (2016) and Zhu et al. (2020) found that tweets related to scientific and conspiracy content (unrelated to environmental issues) displaying more negative emotion get retweeted more than tweets displaying more positive emotion. Similarly, Fan et al. (2014) found that in general, angry messages in particular are more likely to spread on Weibo than joyful or sad ones. More specific to environmental topics, De-Lara et al. (2022) examined the Facebook posts of 10 public figures related to the Madrid Climate Summit of 2019 (also known as COP 25), and found that generally more emotional posts received more interactions from the public than drier, more rational or information-heavy posts. Additionally, in a study of the communication of nine environmental non-governmental organizations (NGOs), Barrios-O'Neill (2021) found tweets related to biodiversity with more negative sentiment received more engagement. Curiously, however, in a study of tweets related to the 2014 Earth Hour campaign on Twitter, Fernandez et al. (2016) found that tweets with positive sentiment generated higher levels of engagement.

Thus, within the experimental research on emotional framing as well as the initial studies of emotional framing of opinion leaders on social media, there is a lack of consensus regarding how we can expect emotions to impact the audiences exposed to it. This discord underscores one of the major challenges underlying this area of research: Emotion is but one factor influencing our attitudes and behavior. Our emotions interact with other processes to contribute to our overall affective state, or mood, which is likely a more consistent predictor of behavior and influence than individual emotional responses (Russell and Barrett, 1996). It is therefore not safe to assume that the response to any given emotional framing will be the same for every person who encounters it (Chapman et al., 2017), and we should rather try to understand the larger context of cognitive processes contributing to ultimate attitudinal and behavioral outcomes, summarized by our mood.

Mood and emotion are sometimes used interchangeably but most psychologists agree that the two are “closely related but distinct phenomena” (Beedie et al., 2005). Parkinson et al. (1996) define mood as “an undirected evaluative mental state which temporarily predisposes a person to interpret and act toward a wide variety of events in ways according to its affective content” (p. 9–10). Watson and Clark (1994) conceptualize mood as a “transient episode of feeling or affect” influenced by internal and external processes, including emotional responses to our thoughts and environment. Gray and Watson (2003) synthesize these definitions into an understanding of mood as “states of mild to moderate intensity that wax and wane gradually over time” (p. 27). They go on to make a distinction between mood and emotion, stating that mood, unlike emotions “cannot be linked to a specific precipitating event or experience, but rather reflect the cumulative effect of multiple inputs (including both internal endogenous processes and external events)” (ibid). Finally, Russell and Barrett (1996) propose the framework in which emotions are pre-conditions for establishing a given mood, and that mood is therefore mediated by the full spectrum of our emotional and cognitive responses to stimuli in our environment, including the information we take in from it and its emotional framing.

Therefore, it could be the case that the disagreement in the literature on emotional framing results from inaccurately assuming a linear path between emotional framing and behavioral responses. For instance, the emotional response to a given stimuli could trigger the formation of different moods in different people depending on how that emotional response in each individual interacts with their other internal processes and predispositions. Thus, it appears that the traditional approach of studying the relations between specific discrete emotions and engagement without accounting for how they interact with other processes may not be sufficient for determining the true effect of emotional framing in environmental communication.

The research presented here seeks to clarify some of this uncertainty. To do so, we first measure positive and negative emotion in the tweets posted by fifty environmental activists over 5 years, the largest sample collected to date. Using this data, we investigate the following questions:

1. How frequently do environmental activists use positive and negative emotion in their posts related to environmental issues on Twitter? How does their use of these features differ within environment- and not environment-related discourse?
2. How does the use of positive and negative emotion relate to the retweet popularity of environment-related activist tweets?

The second part of this research endeavors to see if the results of the Twitter analyzes can be replicated in an experiment in which participants are shown real tweets differing in emotional framing. Additionally, we use test mood as a mediator between the emotional framing and reported action intent. Specifically we ask:

1. Do participants report more action intention upon viewing positively framed or negatively framed tweets related to climate change?
2. Does mood upon viewing the tweets mediate this relation?

As noted in the Introduction, there has been little experimental work using social media posts as communication stimuli. To the best of our knowledge, the only study to do so is DiRusso and Myrick (2021)'s experiment with Instagram posts about plastic pollution. Thus, the combination of the Twitter analysis and the smaller scale experiment using Twitter posts as stimuli contributes to an under-researched line of inquiry by characterizing the roles of emotion and mood in influencing the pathway between environmental activist communication on social media and pro-environmental outcomes.

## 3. Study 1: Activists on Twitter

### 3.1. Materials and methods

#### 3.1.1. Data

In the first study, we examine expressions of emotionality in the communication of environmental activists on the social media platform Twitter. Twitter is a platform that allows users to share written text, images, videos and links to external web pages with their “followers.” These posts are called “tweets.” Users can also share the tweets of other users *via* the “retweet” mechanism which posts the original tweet onto the retweeting user's profile, thereby exposing it to that user's follower network. This is the primary mechanism by which information spreads on Twitter. While there are other forms of interaction on Twitter, including liking and replying to a post, retweets are considered the most reliable indicator of influence spread, or diffusion, on the platform (Cha et al., 2010; Kwak et al., 2010; Boyd and Crawford, 2012; Metaxas et al., 2015).

The sample of activists examined comes from Luo et al. (2020) which used a list of Twitter users, each labeled as either an environmental activist or denier for a language classification task. The list contains 100 users of each category and was originally curated by Wikipedia users. In this paper, we are only interested in the activists who still maintained active profiles at time of collection (January 2021). This resulted in a set of 50 users. We also verified that none of the users were automated “bot” accounts. Further details on this validation process are provided in the Supplementary material.

All original tweets posted by the users on this list publicly and still available from between November 2015 and December 2020 were collected using the Twitter Academic Research API. After removing all duplicates, retweets, and replies, the collection resulted in a dataset of over 510k total posts. This figure is in line with the weekly average tweet rates across the users in the sample. The activity and retweet details of all the accounts are provided in Supplementary Table S1.

To identify the tweets most likely related to environmental issues, an iterative process to identify a set of keywords identifying content related to climate issues was undertaken. This consisted of first searching for tweets containing “climate change,” “carbon,” and “environment” and then examining random samples of tweets not including these terms but indeed related to climate issues in order to identify which words or phrases should also be included. Iterations continued until no additional terms could be identified to reliably expand the classification.

This process resulted in a final set of the following terms: *biodiversity, carbon, clean energy, clean power, climate, climate change, CO*_2_, coal, ecofriendly, emission, environment*, EPA (the abbreviation of the United States' Environmental Protection Agency), *global warming, green, oil, pollution, renewable, solar*, and sustainab*. The asterisks denote the stem of the word for the terms that have multiple forms, e.g., environment vs. environmental and sustainable vs. sustainability. The other terms are already in the stemmed version according to the stemmer available *via* the Gensim package for Python. See Supplementary material for a summary of how we validated the classification approach. This process resulted in the identification of 218k environment-related tweets (43% of the sample).

#### 3.1.2. Analysis

The Linguistic Inquiry and Word Count (LIWC) dictionary was used to extract measurements of positive and negative emotion from each tweet in the full dataset. LIWC employs a bag-of-words dictionary approach to quantify language use in various sociopsychological categories (Tausczik and Pennebaker, 2010). It functions as a software program which takes text as input and returns a set of scores for 92 domains per input. For each domain, LIWC reports the number of words in the input text belonging to a unique dictionary of words defined for each domain. These dictionaries were curated by psychologists, linguists, and sociologists, and then validated externally in experimental studies (Tausczik and Pennebaker, 2010; Pennebaker et al., 2015). Some of the domains pertain to grammatical features of text, e.g., the number of words, verbs, nouns, etc. Other domains refer to various emotions (e.g., sadness, anger, happiness, fear, etc.), cognition, relativity, and social processes. The score for a domain is calculated by comparing the number of words per text corresponding to the domain dictionary to the total number of words in the text, according to the following formula:


(1) $$\begin{array}{l} Score\left(domain_{x},text_{t}\right)=100*\frac{\left|words_{x}\cap text_{t}\right|}{\left|text_{t}\right|} \end{array}$$

Where *x* is a given domain and *t* is in the input text. As discussed in Pennebaker et al. (2015), LIWC has been subjected to rigorous psycholinguistic evaluation across a variety of contexts including news media, social media, professional communication (e.g., email), and casual language (e.g., text messaging). As such, its validity and reliability in detecting grammatical and semantic categories in text is generally accepted by scholars in numerous research fields, and has led to its status as a standard method in linguistic analysis. A few notable examples of research with LIWC include Sharma et al. (2017)'s analysis of abortion discourse on Twitter; Tumasjan et al. (2010)'s construction of psycholinguistic profiles of major figures in the online German political discourse in the run-up to a major election; Pietraszkiewicz et al. (2019)'s investigation of gender-stereotyped use of agency and communion language in job adverts; Dyer and Kolic (2020)'s construction of semantic networks of death and affect in Twitter discourse related to COVID-19; and Tay (2021)'s comparison of the sociopsychological profiles of newspaper coverage surrounding the 2019 Hong Kong protest movement.

For illustrative purposes, Table 1 contains examples of tweets from the environment- and not environment-related subsets scoring highly for each feature. Before conducting the linguistic analysis, the tweets are preprocessed to remove URLs, usernames, punctuation, and stopwords.

**Table 1:** Examples of high scoring tweets from activists per psycholinguistic feature examined in the study.

**Feature**	**Environment-related tweet**	**Not environment-related tweet**
Positive emotion	Lots of ways to lower carbon emissions associated w/ dietary choices. Vegetarian is awesome, but pescatarian (what I am) better than meat, chicken is better than beef. Local food is less energy-intensive, etc. Each of us can find ways to improve consistent w/ our preferences.	Thank you dear women of our lives! Thank you for your wisdom, courage, and strength! #100daysofresistance #WomensMarch
Negative emotion	Do you think it's right that we go on destroying the natural world? A bleached reef is a tragic sight. A desperately tragic sight, particularly if you've seen it before, and you know what it could have been like. #ActOnClimate #ClimateCrisis	Outrageous. Disgusting. Latin America leaders condemn Trump's Mexico wall #ImmigrantRights #CELAC #DumpTrump

Next, two regression models are used to model the relationships between the variables of interests. Tweets are nested within communicators as the model cases are not independent, i.e., the stylistic variability of each user and the fact that they have different numbers of posts and followers. This allowed us to achieve standard error estimations which are robust to non-independence of the tweets' features. Each model includes fixed effects for year, month, and weekday given the strong cyclical trends and high variation over time of retweet engagement. The first year of the dataset (2015) is used as the reference level for year, while the modal values of month (November) and weekday (Wednesday) are used for those variables.

The first model determined if the usage patterns of the psycholinguistic features differed in tweets that are environment-related and those that are not. The second model determined if any of the features are related to higher or lower retweet engagement. Retweet engagement here serves as a proxy of audience support for the content expressed in activist posts. Previous research confirms the utility and validity of singling out retweets as a measure of influence and engagement (see Cha et al., 2010; Kwak et al., 2010; Metaxas et al., 2015; Majmundar et al., 2018. The number of “likes” a post receives could also be used to approximate engagement but this mode of interaction is generally considered weaker than retweets (Metaxas et al., 2015). Further discussion of using retweets as the primary proxy of engagement can be found in the Supplementary material.

In each model, the number of followers each user had at time of collection and the word count of each tweet, excluding usernames and URL links, are included as covariates. Word count is not usually accounted for in Twitter studies, due to the short character limit (280 characters) imposed by the platform. However, it is possible there is some variation in tweet length which could reflect different communicative intentions. Indeed, there is significant variance in length among the tweets in our sample, so it is included here to afford for any impact this variable could have on retweet probability. Follower count and number of retweets are log transformed because they are highly right skewed. Moreover, the regressions used bootstrapped standard errors which are robust to non-normality to further account for the skew in these variables.

Finally, a path analysis is undertaken to determine whether the environment relevance of a tweet is related to the use of the emotional linguistic features, and in turn, if this affects retweet count. To do so, we again use bootstrapping in which a mean indirect effect is computed using a re-sampling method, typically consisting of 5,000 iterations (Shrout and Bolger, 2002). The method outputs *p*-values, confidence intervals, and standard error values for estimations of the direct and indirect effects for the variables included in the model, which are used to interpret the mediation paths.

### 3.2. Results

Before building the models, we check for collinearity in the datasets by examining the correlation between each pair of variables. The results indicate that none of the variables are correlated above the acceptable threshold of 0.85 (Kline, 2005) so we proceed with the modeling. The descriptive statistics and correlation table for each variable are presented in Tables 2, 3, respectively. The scores for each linguistic feature correspond to the average percentage of total words in a tweet corresponding to each feature.

**Table 2:** Descriptive statistics for the variables used in Study 1.

		**Overall (*****N*** **= 510,319)**		**Environment-related (*****N*** **= 218,100)**		**Not environment-related (*****N*** **= 292,219)**	

**Variable**	**Base rate**	**M**	**SD**	**M**	**SD**	**M**	**SD**
Positive emotion	5.48	3.65	6.32	2.88	4.07	4.22	7.13
Negative emotion	2.14	2.19	4.26	2.20	3.92	2.18	4.42
WC	-	20.3	11.2	22.5	10.9	19	11.2
Followers	-	551540 (12.3)	1711547 (1.6)	365447 (12.2)	1038317 ( 0.94)	690432 (12.4)	2065427 (1.14)
Retweets	-	147.56 (2.79)	1749.98 (1.60)	92.3 (2.91)	841.9 (1.47)	188.9 (2.69)	2194.3 (1.68)

For two variables—Retweets and Followers—the descriptive statistics are presented in addition to the logarithmic transformation in parentheses. Constant of 1 has been added to all the transformed variables for scaling reasons.

**Table 3:** Correlation coefficients for the variables used in Study 1.

**Variable**	**2**	**3**	**4**	**5**
1. Positive emotion	–0.106***	–0.124***	0.022***	–0.064***
2. Negative emotion		–0.044***	0.012***	0.056***
3. Word count			0.048***	0.235***
4. Followers_*T*_				0.519***
5. Retweets_*T*_				

Subscript T refers to logarithmic transformations. Constant of 1 has been added to all the transformed variables for scaling reasons.

***p ≤ 0.001.

**Figure 1:**
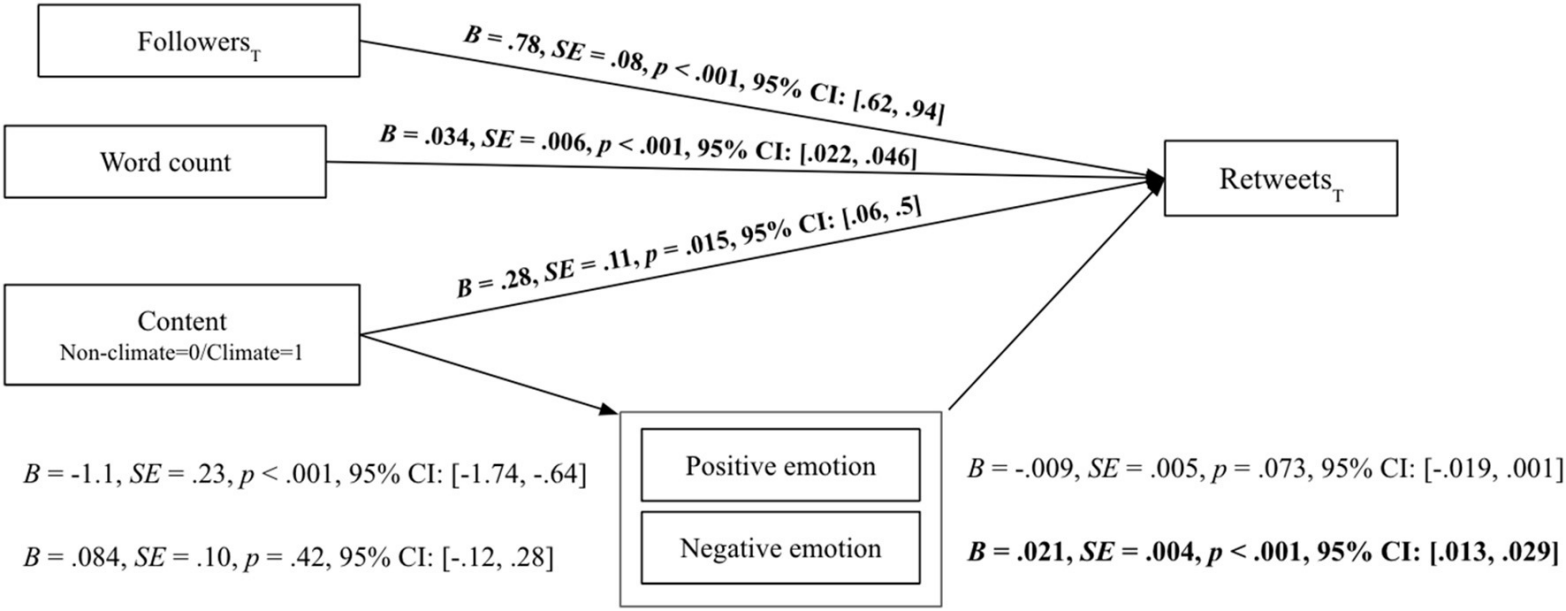
Schematic representation of the models used to determine the direct and indirect effect of followers, word count, content climate relevance, positive and negative emotion on retweet popularity for activist tweets. Unstandardised coefficients, standard errors, *p*-values, and 95% confidence interval for each coefficient are provided on the lines connecting each variable. Variables for followers and retweet count are marked with a subscript T to denote their log-transform.

The distributions of retweets and followers per user are both heavily skewed toward larger values and as such they must be transformed to better approximate the normal distribution. The log-transform works to accomplish this for both distributions so the log of retweet and log of followers are used in all models. Figure 1 visualizes a schematic of the variables and relations modeled. The full output for each model is provided in the Supplementary material.

First, the relation between emotion feature use and climate content relevance is examined. The coefficients of this model are listed to the left of the emotion features in Figure 1. They indicate that positive emotion is used much less in environment-related tweets than in those unrelated to climate issues (*B* = –1.1; *SE* = 0.23; *p* < 0.001; 95% CI: [–1.74, –0.64]), while use of negative emotion is statistically consistent in both subsets (*B* = 0.084; *SE* = 0.1; *p* = 0.42; 95% CI: [–0.12, 0.28]).

Second, all variables, including the log of followers, climate content relevance, tweet length, and the emotion features are used to predict the log of retweets. This model accounted for 34% of the variance of log retweets: R2 = 0.342, *SE* = 0.036, *p* < 0.001, 95% CI = [0.27, 0.41]. As in previous studies, the number of followers was a significant predictor of the retweet count (*B* = 0.78; *SE* = 0.08; *p* < 0.001, 95% CI: [0.62, 0.95]). Tweet length in terms of word count is also positively related to log retweets (*B* = 0.034; *SE* = 0.006; *p* < 0.001; 95% CI: [0.022, 0.046]). Importantly, we also found a significant effect of content climate relevance (*B* = 0.28; *SE* = 0.22; *p* = 0.015; 95% CI: [0.06, 0.5]), suggesting environment-related tweets of activists were more retweeted than not environment-related tweets.

In terms of the emotion features, tweets were more retweeted the more they contained negative emotions (*B* = 0.021; *SE* = 0.004; *p* < 0.001; 95% CI: [0.013, 0.029]), but the less they had references to positive emotion (*B* = –0.009; *SE* = 0.005; *p* = 0.073; 95% CI: [–0019, 0.001]) although the statistical significance of this effect narrowly misses the α = 0.05 significance level.

The path analyzes show that the indirect effects through positive and negative emotion between climate relevance and retweet engagement both amounted to 0. Therefore, there is no evidence to suggest that activists used more or less positive or negative emotion when talking about environmental issues and that this in turn led to higher retweet engagement.

## 4. Study 2: Experiment

The results of the first study indicate that environment-related content tends to be retweeted more when containing more negative and less positive emotionality. In Study 2, we aim to confirm this effect in a controlled experimental setting by focusing on environment-related tweets only and manipulating the emotionality of the presented tweets. In response to such an emotional content participants could express their intention to retweet and more generally their intention to engage in climate action. We expect that in accordance with Study 1, less positive content and more negative content will elicit higher retweet and behavioral intention.

Additionally, we studied a psychological process standing behind the decision to retweet or engage with climate action. As described in the related work, mood is influenced by emotional framing (Russell and Barrett, 1996) and thereafter influences our actions and perspectives (Rucker and Petty, 2004; Barsade et al., 2018; DiRusso and Myrick, 2021), and as such potentially acts as a mediator between information input and behavioral response in the context of environmental issues.

A between-subjects online experiment was conducted in a Qualtrics survey and distributed to Prolific participants in January 2022. Participants were randomly distributed into the positive or negative framing condition. This study was approved by the institutional ethical review boards at the authors' respective universities.

### 4.1. Materials and methods

#### 4.1.1. Participants

An analysis of statistical power (*f* = 0.25, α = 0.05, 1-β = 0.80) specified the target sample size as 128 participants. In order to allow correction for low data quality based on attention checks, we decided to collect 200 participants. The participants received £11.36 per hour for agreeing to take the survey, which took the participants on average 6.2 min to complete.

We exclude 24 of the participants on the grounds of failing manipulation checks. These checks are further described in subsequent paragraphs. Seven others are also excluded because they indicated they do not believe that action to mitigate climate change is necessary. We do not repeat the analysis with these users because the target group only includes people who would be most likely to attend to and engage with environmental activist content, i.e., resembling those who engaged with the tweets analyzed in Study 1.

After these exclusions, 169 participants remain. Of these, 75% identify as female, 23% as male, 2 participants prefer not to say, and 1 elected to provide their own description. The age of the participants ranges from 18 to 68, with an average of 35 years and a standard deviation of 11.7 years. Most of the respondents (38%) had attained at least a bachelor's degree. Additionally, the majority (55%) of respondents report a left-leaning political orientation, with a smaller yet sizeable proportion (30.8%) reporting neither left- nor right-wing. Regarding social media use, 94.1% of participants reported having used at least one social media platform in the last month. Of these, 75.1% reported using social media multiple times per day.

#### 4.1.2. Procedure

Upon recruitment to the experiment, participants were shown a description of the study, including its purpose, and further information on the study's voluntary nature, the confidentiality of responses, and data protection protocols along with Article 13 of the European Union's General Data Protection Regulation (GDPR). Participants were then asked to consent to join the experiment. Participants were then allocated randomly to the positive or negative emotional framing condition. Depending on this allocation, participants were shown a set of tweets and asked to review them. Immediately after viewing the tweets, participants answered items about their mood (valence and the state of arousal), position on the necessity of climate action, likeliness to retweet the tweets they saw, and their likeliness to participate in a battery of actions to support their stance on climate action. The survey then asked participants to write a slogan to encourage participation with climate action. The participants were made aware that providing a slogan was completely optional.

Then, participants were asked to answer a series of demographic questions (the variables named in the description of the participants). Finally, the survey implemented checks to determine the effectiveness of the manipulation and how well participants had been paying attention to the questions of the experiment (Hoewe, 2017). First, a question informed the participants that they had reached the end of the experiment and that they should select “No” to proceed. This was done to catch any participants who were not carefully reading the questions.

Second, participants were asked to recall the tweets they had been shown and to rate their tone on a seven-point scale Likert from “very negative” to “very positive.” This served as a manipulation check and was done to ensure that participants had perceived the tweets in accordance with their condition. Third, they were shown a list of tweets and asked to select the ones they had been shown. This was done to validate meaningful engagement with the experiment. Participants who failed to answer “No” on the first question, correctly report the tone of the tweets, or identify the tweets they had seen were excluded.

#### 4.1.3. Stimuli

Each experimental condition included a set of three tweets. The text of the tweets was either directly copied or lightly paraphrased from tweets in the dataset collected in Study 1 which scored highly on either positive or negative emotion. They tweets were selected, and lightly edited when necessary, to be as similar between the conditions as possible in terms of length and strength of sentiment valence. The three tweets were shown to the users all together.

The images of the tweets were generated artificially using the website TweetGen. We did this so that we could standardize the number of retweets, likes, and comments received by the tweets pairwise between conditions, thereby controlling for any bias resulting from impressions of retweet count popularity (Taylor et al., 2022). We also obscure the place where the user's name and Twitter handle would be to prevent any bias resulting from affinities or disaffinities for particular activists. Limitations deriving from this decision are discussed later in the paper.

*The text of the three tweets in the positive condition include:*

- Talking about #ClimateChange and #ClimateAction as “opportunities” can inspire hope.
- #ClimateAction is a blessing not a burden! More jobs, better jobs; Inclusive, robust economies; Lower healthcare costs; Better security!
- Transitioning to renewable energy to flight #ClimateChange strengthens national security and is a source of innovation, jobs, and wealth.

*Meanwhile, the three tweets in the negative condition include:*

- The inaction of our governments on the climate emergency is a threat to everyone. It is infuriating!
- Depression, anxiety, post-traumatic stress disorder, domestic & substance abuse all tend to go up in the aftermath of a #ClimateDisaster.
- #ClimateChange posts a critical national security threat and is amplifying many hazards and dangers for all of us.

#### 4.1.4. Measures

Mood response was measured using the nine-point Self-Assessment-Manikin scales for valence and arousal as presented in Lang (1980). The two scales comprise an established picture-based methodology for the extraction of people's emotional states. Each scale consists of 9 illustrations, each depicting a caricature manifesting emotional states. They range from sad (value 1 on the scale) to happy (value 9 on the scale) in the scale of valence and from calm (value 1 on the scale) to excited (value 9 on the scale) in the scale of arousal. Immediately after viewing the tweets, participants were asked to indicate which figure best represents their current emotional state.

To determine the participants' stance on the necessity of climate action, they were first asked to think about human-driven climate change. They were also told that it is the center of a heated debate. The question text then informed the participants that the authors of the tweets they had just seen express the need for climate action to protect the environment, while others do not think climate action is necessary. They were then asked to indicate their position within this debate using a seven-point Likert scale with options ranging from “definitely unnecessary” to “definitely necessary.”

The primary dependent variable of our analysis is reported intention to partake in collective action after viewing the tweets. The belief-aligned collective action scale presented in Cervone et al. (2021) is used. Examples of actions on the scale include: “*I would carry out research to learn more about possible actions I can take to promote my position”* and “*I would attend a rally, a march, or a protest to assert my position.”* Additionally, to make the Study 2 coherent with Study 1, we added to the scale a question on likelihood of retweeting the presented tweets. Participants are asked to rate their likelihood of engaging with each of the actions using a seven-point Likert scale with options ranging from “very unlikely” to “very likely.” The Cronbach's alpha score for the items is 0.905, indicating a very good level of internal consistency.

#### 4.1.5. Analysis

The results were analyzed in three stages. First, a one-way analysis of variance (ANOVA) is used to determine whether the experimental conditions differed with respect to the mood they elicit in participants. Then, another ANOVA is carried out to determine whether the experimental conditions differed with respect to elicited action intention. Finally, path analysis is used to measure the total, direct, and indirect effects of the experimental conditions and mood on reported collective action intention.

#### 4.1.6. Results

In order to see whether the two experimental conditions differed with respect to elicited mood we conducted a one-way analysis of variance (ANOVA). Indeed participants in the positive emotion condition had a more positive mood (*M* = 5.84; *SD* = 1.34) than participants in the negative emotion condition [*M* = 3.94; *SD* = 1.26; *F*_(1, 167)_ = 89.07, *p* < 0.001; η^2^ = 0.35]. Arousal on the contrary, was not affected by the experimental manipulation [*F*_(1, 167)_ = 0.43, *p* = 0.51; η^2^ = 0.003], so participants in the positive (*M* = 4.66; *SD* = 1.48) and negative condition (*M* = 4.49; *SD* = 1.87) were similarly aroused by the content of messages.

Second, we examined the direct relationship between the two experimental conditions and reported collective action intention. The latter was not affected by the experimental manipulation [*F*_(1, 167)_ = 2.04, *p* = 0.16; η^2^ = 0.01], so participants in the positive (*M* = 3.76; *SD* = 1.18) and negative condition (*M* = 3.47; *SD* = 1.50) were similarly keen on taking a collective action after seeing the messages.

**Figure 2:**
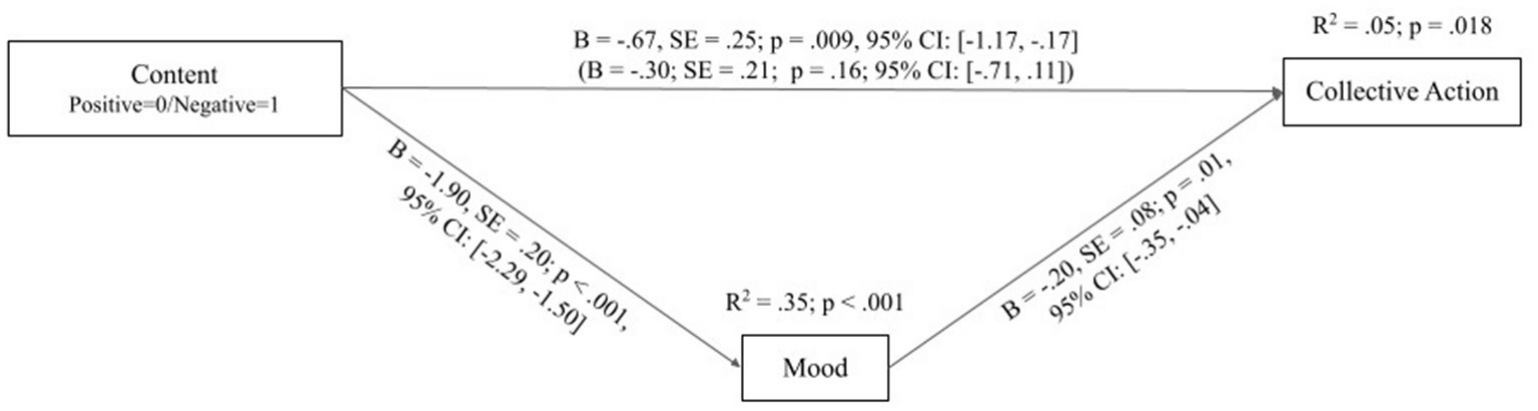
Visualization of the mediation analysis showing the direct relationship between content and collective action, along with an indirect relationship between the two mediated by mood. The total effect between content and collective action is given in parentheses below the direct effect on the arrow connecting the two variables in the diagram. The coefficient of the direct relationship is negative while the effects of the indirect path (content to mood and mood to collective action) combine to yield a positive coefficient.

To see whether the manipulation affected the valence of mood, which in turn had an effect on collective action intentions, we conducted the mediation analysis presented in Figure 2. This analysis tested the direct and indirect relationships between content framing, mood, and collective action intent. There are two main findings.

First, the path analysis revealed a significant direct negative relation between negative content framing and collective action intent, suggesting that seeing the negatively framed tweets led to lower reported intent to engage. This result appears to contradict the outcome of Study 1, which suggested that content with less positive and more negative sentiment may increase action tendency measured through retweet engagement. However, the path analysis also revealed a significant *indirect* effect of framing through mood on action intention: Negative framing decreased reported mood which *increased* reported collective action intent. This effect amounted to 0.37 with a bootstrapped 95% confidence interval of 0.06 to 0.71 and is congruent with Study 1.

Therefore, including mood as a mediator suggests a final significant direct positive effect of negative content framing on collective action. Given the insignificant total effect between negative content framing and collective action (given in parentheses below the statistics of the direct effect on the arrow connecting the two variables in Figure 2), such an increase in magnitude of the relationship between a predictor and an outcome indicates statistical suppression (also: inconsistent mediation, MacKinnon et al., 2000). Specifically, within a mediation model, a suppression effect is present when the direct and mediated effects of an independent variable on a dependent variable have opposite signs.

In line with that, the results imply the presence of two opposing processes in the model. On the one hand, seeing negative tweets increases willingness to engage because it depresses mood (the indirect pathway), potentially increasing a sense of urgency to act. On the other hand, it decreases engagement in collective action (the direct pathway) potentially *via* another mechanism yet to be identified. These results and possible explanations for them are further discussed in the next section.

## 5. Discussion

The results of both the Twitter study and the experiment suggest that framing with negative emotion may be more effective than positive emotion in generating engagement within individuals who are likely to be concerned about environmental issues. The primary result of the Twitter analysis is that environment-related content posted by activists is retweeted more when it contains more negative emotion and less positive emotion. This result converges with previous studies for other discourses on social media which found preferential engagement for content with higher negative sentiment (Soroka and McAdams, 2015; Zhu et al., 2020).

One potential explanation of this result may be that more negative messages from activists are more effective at eliciting support *via* retweet as a signal of participation with environmental *clicktivism* (Rotman et al., 2011; George and Leidner, 2019; Morozov, 2019). However, the findings of the Twitter study are unable to reveal whether this bias toward negativity extends beyond retweet engagement and into offline behavior. Therefore, the results raise questions as to whether exposure to environment-related content on social media with more negative content motivates audience members to support the environmental movement offline through collective action. This question was investigated in Study 2.

The results of the experiment reveal a duality in the effect of emotional framing on action intention. When directly modeling the relationship, participants exposed to messages with negative emotion were less likely to report higher likelihood of engaging with climate action. However, at the same time when mood was used to mediate the relationship, we find that messages with negative emotion elicited a more negative mood in participants and they therefore reported to be more likely to engage with the offline action. The contradiction between the direct and indirect effects is evidence of a suppression effect of mood on the relationship between emotional framing and action intention. The indirect effect is similar to the results obtained in Study 1, where negative content elicited more engagement through retweeting. However, the direct effect goes in the opposite direction, showing that negative emotional framing can also inhibit reported readiness to act.

These results make clear that studies of emotional framing in environmental communication should not assume a direct linear relationship between emotional framing and action outcomes. Researchers need to incorporate additional processes which may mediate the potential of a given emotional frame to stimulate action intent. If we had not included mood in our model, the coefficient on the direct relationship between emotional frame and action intent would have led us to conclude that the higher engagement with messages containing more negative emotion observed in the Twitter study could not be replicated in a smaller scale experiment offline. In which case, it would have suggested that engagement trends on Twitter may not be able to tell us anything meaningful about offline engagement in this context. Instead, including mood in the model suggests that people may be more likely to engage with more negative content about climate change because it triggers a negative emotional state.

As summarized in the literature review, most work on emotion in environmental communication has investigated how framing messages with emotion makes participants feel about the legitimacy and risks of climate change or affects motivation for taking action. These studies have generally found mixed results regarding positive vs. negative emotional framing leading to higher levels of reported engagement with climate action. The lack of consensus in these results likely stems from the implied assumption that emotion alone governs our responses to environmental communication, leading to the exclusion of other mechanisms influencing the pathway between content framing and outcome variables. Our results show that it is imperative to explore and understand the mechanisms which lead to different, and perhaps competing, behavioral responses to the same message. Mood is undoubtedly but one of several mechanisms governing this pathway. It is also likely that emotional framing is not the only type of communication framing with the power to influence mood. While previous studies have examined different dimensions of framing in environmental communication (e.g., specific vs. abstract advice, local vs. global consequences, collective vs. individual responsibility) and their effects on reported action intention, none have accounted for the psychometric mechanisms or processes which mediate this pathway.

We have been discussing the experiment results mostly in terms of the impact of negative framing, but we could also describe them in terms of the positive condition: Seeing positive tweets facilitated engagement in collective action, however at the same time it decreased willingness to engage because it elevated mood. One potential explanation of the results from this perspective comes from relative deprivation theory (RDT). The traditional focus of RDT is when, how, and why people subjectively experience unjust advantages, and how this perception can lead to collective action (Stouffer et al., 1949; Merton and Lazersfeld, 1950; Runciman, 1966; Pettigrew, 1967; Crosby, 1976; Crosby and Gonzalez-Intal, 1984; Folger, 1987; Walker and Smith, 2002). RDT began from the observation that objective collective deprivation could not predict collective action outcomes. Instead, the subjective experience of collective deprivation could. As such, RDT has been used to determine what social comparisons foster collective action and which do not (Smith and Ortiz, 2002), as well as whether affective components of group-based deprivation impact motivation for collective action (Guimond and Dubé-Simard, 1983; Tyler and Smith, 1998).

These investigations of RDT have led to the understanding that group-based anger toward a collectively-felt grievance or injustice is a powerful motivator for collective action. Therefore, because the positively-framed messages induce a positive mood about climate change instead of a sense of anger toward a collective grievance, the messages are not uniformly successful at motivating collective action. Rather, the positively-framed messages might elicit a kind of passivity, or a divorcing of individual responsibility from the problem because the messages give the idea that things are not so bad or that someone else is already taking care of it (van Zomeren et al., 2019). Adding further support to this explanation, the work of Bloodhart et al. (2019) and Iniguez-Gallardo et al. (2021) demonstrate that many people who actively attend to environmental issues have strong negative emotions associated with them. Thus, it could be the case that when they then encounter communication which lifts their mood, it may reduce their sense of urgency to act.

Further research should probe this relationship between positivity and collective action, while also taking into consideration the different narratives and topics that could be discussed. For example, talking about climate action solutions in a positive manner without emphasizing how or why individuals and communities can get involved may be one reason positive messages are less galvanizing. The confluence of emotion and agency is extremely important and needs to be further examined in order to identify ways of formulating more effective and engaging environmental communication.

Finally, it is clear that our model does not capture everything governing the pathway between information framing and collective action intent. For example, it is likely that the direct pathway we observe is mediated by some other mechanism(s), which the mood pathway cancels out (or vice-versa) in terms of outcomes for collective action intent. Future research should endeavor to identify and test these other mechanisms.

## 6. Limitations

The work contains limitations. First, we only analyze the text of each tweet, excluding any videos, images, or links that might be associated with them. This means we lose some information conveyed by these attachments. Moreover, while LIWC continues to be one of the most popular choices for broad computational linguistic analysis, the tool is not without shortcomings. LIWC does not enable us to detect higher-level semantic features such as irony and sarcasm. Furthermore, it is not able to account for context-related features of language which are not commonly detectable with a lexicon-based approach.

Additionally, the external validity of research using Twitter data is inherently limited because it is not representative of any given demographic population, nor of the full social media discourse on environmental issues. Moreover, we only focus on fifty activist accounts. These are not representative of the full scope of environmental activists on social media. Nonetheless, these users constitute a sample of prominent opinion leaders in online environmental activism. It is therefore still useful to study them to get an initial sense of the general trends in emotional framing that environmental activists use in their communication on Twitter. They present an initial benchmark for the link between emotional framing and retweet engagement for future work to expand upon with investigations of other users, perhaps on different platforms, and with other types of interactions.

While the Twitter study seeks to identify the correlation between emotion in tweets and retweet engagement, it does not take into account non-content related variables of the tweets, besides each user's follower count, and how these might also affect engagement. Acknowledging that the study is not able to test for all possible variables that may account for user engagement with online content, it does provide valuable insight into the significance of a range of psycholinguistic features on social media.

Moreover, both the Twitter study and the experiment were conducted using English speakers and English text. Therefore, we were not able to provide an analysis of communication published in other languages. Future research could address this linguistic bias by repeating this analysis for another set of activists who produce content in diverse languages and geographic regions.

Turning to the experiment, the insights we are able to offer are limited to reported behavior. People are known to report themselves as more environmentally friendly than they actually are in real life (Kormos and Gifford, 2014). Thus, we must acknowledge that the participants in our experiment may have over-reported their intention to engage in collective action. However, this is a limitation of all research in environmental communication using one-off experiments, which to date, makes up a bulk of the field. A longitudinal study of participants controlling for the psycholinguistic features of the environmental communication to which they are exposed over time would be ideal but also highly resource intensive. As such, one-off experiments give us the most efficient glimpse into the human psyche, providing useful data upon which subsequent studies can build.

Another concern is the extent to which the observed effects of the experimental conditions result purely from the positive or negative framing of the tweets. Although the tweets were selected based on the dominance of positive or negative emotional valence over other psycholinguistic features, it is still possible that connotations of the content differed among participants. It is also possible that references to certain topics in the tweets resonated unevenly with participants, eliciting different reactions that had little to do with the framing manipulation. Additional piloting of the tweets could have helped to identify the existence of these potential confounding variables. Nonetheless, this is a trade-off of using real tweets and not designing content to be as “sterile' as possible with respect to potential confounding variables. At the risk of increasing the presence of potentially confounding variables, we test tweets manifesting the true framing tendencies of environmental activists thereby increasing the external validity of the measures.

Moreover, while the tweets are presented to the users in the same style that they would encounter the messages on the platform itself, the fact remains that they are viewing these tweets within an experiment, a context which is obviously different to how they would encounter the tweets in real life. This is also a limitation of most environmental communication studies in which measuring the effect of a specific frame manipulation is desired. The tweets are also presented to the participants with the username and profile photo obscured. This was done to reduce the chance of inducing reactions to the tweets that might stem less from the emotional framing of the tweet, and more from the stature of the particular activist or opinions the participants may have about the activist. We could not control for participants' awareness of specific activists, thus identity effects were beyond the scope of this experiment. However, they have been demonstrated in previous work on Twitter to have significant effects on engagement (Taylor et al., 2022). Therefore, it would be interesting to attempt to isolate the effects of activist identity on engagement. Moreover, there could also be interactions between identity and emotional framing that future work should consider examining.

Regarding the sample, we only include participants in the analysis who reported to already be convinced of the danger of environmental issues and the need to act. Therefore, the results do not generalize to people who are more undecided or simply less likely to be paying attention to environmental activism, nor those who might outright deny environmental issues as real phenomena. Moreover, the sample is heavily biased toward participants identifying as female. This is potentially problematic as there is evidence suggesting that participants identifying as female have stronger pro-environmental attitudes and behaviors than participants identifying as male, and that this tendency stems from the former demonstrating higher levels of social affiliation and responsibility (Zelezny et al., 2000). Moreover, additional studies have found gender differences in various contexts of emotional regulation and mood management (see Nolen-Hoeksema, 2012 for a review). Thus, it is possible that the results of the experiment are skewed by the gender bias in the participant sample. Additional experimentation should be conducted to ascertain the extent to which the framing-mood-behavior pathway found here generalizes across genders, and/or to determine what gender differences exist.

## 7. Conclusion

In conclusion, this work contributes to the literature of online environmental communication by being one of the first to investigate the relationship between the use of emotional framing by environmental activists on social media and audience engagement. To date, the literature has found mixed results for how we can expect emotional framing to impact how we feel about environmental issues and our likelihood to engage with collective action. The results of the Twitter study support the media negativity bias: people tend to engage more with content when it contains more negative emotion. The results of the experiment provide additional insight into one mechanism—mood—which may underlie the Twitter results: Negative emotion elicits a negative mood which then increases reported action intent. Meanwhile, we also find evidence for another mechanism having the opposite effect (lowering action intent), which appears to interfere with the mood mechanism. These results contradict what previous work has found suggesting a positive effect of positive emotions on engagement with environmental activism.

More work needs to be done to understand why this is the case and what this other mechanism might be. One way to begin could be by examining the impact of additional psycholinguistic features, specific narratives, and content format on action intent. It could be possible that certain narratives when paired with certain emotions in a specific format elicit different mood and action intention responses than other combinations. While there may not be a single “silver bullet” formulation for environmental communication, it is clear that social media can and will play a significant role in the attempts of activists to mobilize more people to take climate action. Further research into what works for which demographics will assist in better understanding how activists can calibrate their activism media to optimize engagement both on- and offline.

## Data availability statement

The raw data supporting the conclusions of this article will be made available by the authors, without undue reservation.

## Ethics statement

The studies involving human participants were reviewed and approved by SWPS University of Social Sciences and Humanities, Faculty of Psychology in Warsaw; University of Oxford, Social Sciences and Humanities Interdivisional Research Ethics Committee. The patients/participants provided their written informed consent to participate in this study.

## Author contributions

MS was responsible for the initial conceptualization of the project, Twitter data collection and analysis, and drafting of the experimental materials. MW and MF deployed the experiment and analyzed the experimental data. MS, MW, and MF drafted the manuscript. RG contributed to the literature, framing, and revision of the manuscript. All authors approved the submitted version.
