# Supplementary material for: Emotional framing in online environmental activism: Pairing a Twitter study with an offline experiment
Source: Front Psychol. 2023 Jan 30;13:1099331. doi: 10.3389/fpsyg.2022.1099331 (PMC9928158; doi:10.3389/fpsyg.2022.1099331)
Supplement: Supplementary file 1 [file Data_Sheet_1.PDF]

Supplementary Material

1 ACCOUNT VETTING

Account vetting We begin with a list of fifty climate change activists and take the following steps to investigate whether or not they are indeed non-robot accounts. We manually evaluate the Twitter profiles of every account on the list to make sure none are "bots" or automated accounts. This process involves first passing each profile through the Observatory on Social Media's (OSoMe) Botometer to detect potential bot accounts. The Botometer is an artificial intelligence tool that compares a given Twitter account against a machine learning model trained on a labelled dataset of accounts known to be bots and assigns a score based on the likelihood that the account in question is a bot.[1]

While the Botometer has been independently validated by the Pew Research Center (Wojcik et al., 2018) and social media research (Varol et al., 2017), it cannot guarantee perfect classification. Thus, we further investigate all accounts that the Botometer scored within the top 10% of users with the highest estimation of bot status. This included all users with a bot likelihood score of 3.5 of 5 and above. These accounts include an English actress, the account for a Yale University magazine, two Canadian academics, a prominent Australian climate journalist, and multiple prominent climate activists. Many of these accounts are actually verified by Twitter and as such are vetted to make sure they are authentic.[2] For the others, we could not find evidence to suggest that these accounts are artificial or otherwise not who they claim to represent. Thus, we conclude that the algorithm mistook these users.

Moreover, fourteen of the fifty accounts on the list of activists are not representing a single person, but rather an organisation or collective of multiple individuals, e.g. Oxfam GB and Greenpeace UK.[3] These accounts could be maintained by more than one actor, and that distinguishes them from individual accounts. Such accounts may have a greater capacity to create content and therefore be more active than individual accounts. At the same time, we cannot exclude the possibility that individual activists also rely on assistance when running their personal accounts. There may be a large variety in the ways in which content for social media is produced behind the scenes within both individual and collective accounts.

In any case, we control for posting frequency and number of followers when modelling retweet engagement to account for potential biases resulting from imbalances in the distribution of post volume per user. It is also important to highlight that the focus of this study is not to identify the relation between frequency in posting behaviour and user engagement, but primarily to identify the psycholinguistic tools employed by those accounts in the content they publish, and how this usage resonates with the experimental literature and correlates with retweet engagement. We are not concerned with what the usage of the psycholinguistic features tells us about the activists themselves, but about how the usage is perceived by the users who interact with their content.

Table S1 shows the total number of tweets and retweets for each of the users in the dataset.

[1] https://botometer.osome.iu.edu/faq

[2] https://help.twitter.com/en/managing-your-account/twitter-verified-accounts

[3] For the accounts in the list, the distinction of individual versus organisation is entirely transparent for the accounts on the list, as the biographies provided make plain whether an account is an individual, collective or an organisation. There are none that fall into a questionable status.

Table S1. Username, number of tweets, and sum of retweets for each account included in the sample. Accounts are organised in the table in decreasing order of tweet count. Accounts with the top 10 most retweets are shown in bold for emphasis.

Username	*Tweets*	*Retweets*	*Username*	*Tweets*	*Retweets*
SierraClub	38517	918620	**MarkRufflao**	**8019**	**8219176**
ClimateHome	24002	100463	PaulEDawson	7584	550998
cathmckenna	23175	449469	**CarolineLucas**	**7551**	**3074254**
guardianeco	23003	757668	LeoHickman	7046	126018
NRDC	21507	778247	EcoSenseNow	7033	642578
MichaelEMann	20565	552026	sunrisemvmt	6166	970689
EcoWatch	20475	467762	dwallacewells	5966	265467
Treehugger	18973	160138	**GeorgeMonbiot**	**5751**	**1706603**
grist	17896	201289	dpcarrington	5671	111518
tveitdal	17741	674460	MaryHeglar	5060	164648
DrShepherd2013	17131	157427	oxfamgb	5014	122000
James_BG	16637	97349	Amelia_Womack	4876	194875
**EricHolthaus**	**13386**	**1705911**	**AOC**	**4747**	**30463907**
EnvDefenseFund	13216	192897	**ChrisGPackham**	**4499**	**1321073**
350	13085	625050	drvolts	4210	390918
Earthjustice	12577	518871	NaomiKlein	3722	1041407
RichardMcLellan	11570	95316	bradplumer	3708	87896
CarbonBrief	10920	92334	KHayhoe	3367	346684
**MikeHudema**	**10470**	**1381195**	JayInslee	3098	521918
Yale360	10053	273760	Jamie_Margolin	2481	181885
UNFCCC	9788	889783	**GretaThunberg**	**1411**	**6064854**
ExtinctionR	9238	603563	Ed_Miliband	1194	964985
**billmckibben**	**9017**	**2086188**	algore	1026	449524
ErikSolheim	8554	875843	BillNye	5714	769415
GreenpeaceUK	8318	388630	**LeoDiCaprio**	**591**	**1505864**

2 IDENTIFICATION OF CLIMATE-RELATED TWEETS

To evaluate the precision and recall of our approach to identify climate-related tweets in the sample, we took random samples of 100 climate-tagged and 100 not-climate-tagged tweets and manually evaluated their climate relevance. The results are satisfactory: all of the climate-tagged tweets were correctly identified as climate-related, while 94 of the not-climate-tagged tweets were actually not climate-related.

Examples of these false negatives include:

“The ocean between Cape Cod & Nova Scotia warmed 3.6F in under 10 years. These rising ocean temps are forcing fish to abandon historic territories and move to cooler waters.”

“Future #pandemics are on the horizon if mankind does not stop its rapid destruction of nature #Coronavirus”

“Nuclear power can be, and should be, one major component of our rescue from a hotter, more meteorologically destructive world.”

Each of these indirectly imply criticism of anthropogenic climate change by making reference to examples of threatening consequences. They do not contain keywords or terms that could be adopted into the set of search terms to increase identification of climate-related posts without the risk of incurring false positives.

3 USING RETWEETS AS PROXY OF ENGAGEMENT

The strength of engagement reflected by retweet count is distinct from that which is reflected by other types of interaction with individual tweets afforded by the platform, e.g., likes and replies. Lipsman et al. (2012) suggest that retweets are more salient symbols of engagement than likes because while the latter indicate appreciation and signal agreement with the content conveyed in the tweet, the act of retweeting expresses

more commitment to the content and willingness to spread it. Furthermore, Kwak et al. (2010) and Cha et al. (2010) argue that replies and mentions are used more often to engage users in conversation than to reflect an inclination to engage with and spread the communicated information.

Returning to retweets, it is possible that a user might retweet an activist's tweets to ridicule or disagree with it, especially given the option to make a "quote retweet", i.e., a retweet with commentary. However, my design is limited to the metadata available from the tweets of each user in the sample. This includes the total like count, retweet count, and reply count, but does not distinguish normal from quote retweets. Therefore, I am unable to distinguish retweets signalling positive engagement with the activists from that which may be seeking to disagree with or degrade them. In order to distinguish these two forms of retweet engagement, it would be necessary to track down each retweet of each tweet in the sample. This was unfortunately beyond the scope of the study. It is not yet known how frequently malign quote retweeting occurs with tweets from environmental activists, and therefore presents an intriguing avenue for future research.

An additional critique of using retweets as a proxy of engagement is not unique to retweets in particular as a mechanism of interaction and influence, but to all forms of engagement with activism online. Primarily, the study of online engagement at the level of counts of interactions, without ways of probing how this engagement affects individuals beyond their online activity, cannot distinguish between sincere movement support and what has come to be known as "clicktivism" (George & Leidner, 2019). The term has been used to describe participation in online activism which requires little effort or commitment, e.g., retweeting or signing online petitions. Critics of clicktivism argue that such actions on their own are insufficient for bringing about substantive developments (Bozarth & Budak, 2017; Gladwell, 2011; Morozov, 2011).

While it is true that not all participation with online activism reflects the same level of commitment to and engagement with the broader movement, it is also hard to deny the impact that the cumulative mobilisation achieved by online social movements has had on precipitating real-world change (Han & Ahn, 2020; Lee & Murdie, 2021; Li et al., 2021; Tufekci, 2013; Tufekci & Wilson, 2012). Thus, it is clear that even if we cannot determine the precise character and underlying motivations of interactions with online activism, they are still important to study as components of the success or failure of online social movements.

4 REGRESSION MODEL OUTPUT

The following applies to all models: Retweet and follower count are log transformed; reference level for year variable is 2015 (first year in dataset); reference level for month variable is November (mode); reference level for weekday variable is Wednesday (mode).

4.1 Model relating climate relevance to positive emotion presence

Table S2.

	Estimate	S.E.	Est./S.E.	Two-tailed p-value
CLIMATE	-1.100	0.228	-4.821	0.000
FOLLOWERST	0.124	0.141	0.883	0.377
WORD COUNT	-0.081	0.029	-2.791	0.005
2016	0.211	0.126	1.674	0.094
2017	0.185	0.204	0.906	0.365
2018	0.921	0.579	1.590	0.112
2019	1.061	0.708	1.497	0.134
2020	0.942	0.604	1.560	0.119
MON	0.014	0.040	0.359	0.719
TUES	-0.028	0.033	-0.832	0.406
THURS	0.015	0.028	0.549	0.583
FRI	0.026	0.050	0.523	0.601
SAT	0.327	0.154	2.124	0.034
SUN	0.367	0.150	2.447	0.014
JAN	-0.344	0.113	-3.040	0.002
FEB	-0.120	0.050	-2.411	0.016
MAR	-0.204	0.086	-2.370	0.018
APR	-0.109	0.109	-0.995	0.320
MAY	-0.125	0.128	-0.973	0.330
JUN	-0.123	0.099	-1.235	0.217
JUL	-0.272	0.125	-2.182	0.029
AUG	-0.296	0.114	-2.599	0.009
SEPT	-0.275	0.073	-3.759	0.000
OCT	-0.153	0.102	-1.502	0.133
DEC	0.145	0.072	2.012	0.044

Dependent variable is positive emotion presence. Log likelihood = -4198164.687. AIC = 8396423.373. BIC = 396947.084. Sample-sized adjusted BIC = 8396797.716. R-squared = .028 (SE = .011, p = .013).

4.2 Model relating climate relevance to negative emotion presence

Table S3.

	Estimate	S.E.	Est./S.E.	Two-tailed p-value
CLIMATE	0.084	0.104	0.804	0.421
FOLLOWERST	0.056	0.072	0.778	0.437
WORD COUNT	-0.021	0.007	-2.962	0.003
2016	-0.073	0.067	-1.092	0.275
2017	0.170	0.100	1.703	0.089
2018	0.177	0.111	1.592	0.111
2019	0.234	0.160	1.459	0.144
2020	0.213	0.148	1.436	0.151
MON	-0.018	0.023	-0.748	0.455
TUES	-0.005	0.021	-0.245	0.807
THURS	-0.030	0.025	-1.174	0.240
FRI	-0.058	0.035	-1.673	0.094
SAT	-0.083	0.074	-1.119	0.263
SUN	-0.067	0.053	-1.262	0.207
JAN	0.099	0.063	1.571	0.116
FEB	0.047	0.048	0.979	0.328
MAR	0.069	0.053	1.299	0.194
APR	0.031	0.048	0.650	0.516
MAY	-0.035	0.050	-0.707	0.480
JUN	0.054	0.051	1.065	0.287
JUL	0.125	0.049	2.556	0.011
AUG	0.076	0.049	1.548	0.122
SEPT	0.050	0.055	0.898	0.369
OCT	0.154	0.041	3.753	0.000
DEC	-0.065	0.049	-1.318	0.188

Dependent variable is negative emotion presence. Log likelihood = -1684517.336. AIC = 3369100.672. BIC = 3369468.384. Sample-size adjusted BIC = 3369363.508. R-square = .003 (SE = .002, p = .068).

4.3 Model relating emotion presence to retweet engagement

Table S4.

	**Estimate**	**SE**	**Est/SE**	**Two-tailed p value**
CLIMATE	0.276	0.114	2.423	0.015
FOLLOWERST	0.775	0.078	9.916	0.000
POSEMO	-0.009	0.005	-1.791	0.073
NEGEMO	0.021	0.004	5.292	0.000
AFFILIATION	-0.006	0.004	-1.614	0.106
AG_EMPOWER	0.000	0.015	-0.027	0.978
FOCUSPAST	0.007	0.006	1.328	0.184
FOCUSPRESENT	0.003	0.005	0.624	0.533
FOCUSFUTURE	-0.005	0.004	-1.460	0.144
WORD COUNT	0.034	0.006	6.019	0.000
2016	0.056	0.057	0.977	0.329
2017	0.198	0.128	1.543	0.123
2018	-0.256	0.139	-1.839	0.066
2019	-0.113	0.151	-0.747	0.455
2020	-0.197	0.162	-1.213	0.225
MON	0.094	0.015	6.409	0.000
TUES	0.016	0.009	1.841	0.066
THURS	-0.006	0.013	-0.492	0.623
FRI	0.032	0.022	1.480	0.139
SAT	0.327	0.062	5.273	0.000
SUN	0.368	0.073	5.007	0.000
JAN	0.098	0.035	2.775	0.006
FEB	0.072	0.041	1.747	0.081
MARCH	0.117	0.042	2.812	0.005
APR	0.054	0.039	1.370	0.171
MAY	0.030	0.037	0.809	0.419
JUN	0.041	0.039	1.034	0.301
JUL	0.050	0.035	1.445	0.149
AUG	0.060	0.037	1.625	0.104
SEPT	0.087	0.033	2.624	0.009
OCT	0.053	0.021	2.476	0.013
DEC	-0.060	0.021	-2.928	0.003

Dependent variable is log retweet engagement. Log likelihood = 5882862.488. AIC = -11765644.977. BIC = -11765199.265. Sample size adjusted BIC = -11765326.387. R-squared = .342 (SE = .036, p ¡ .001).

4.4 Path analysis relating climate relevance, emotion presence, and retweet engagement

Table S5. Total and indirect effect between climate relevance, negative emotion, and log retweet engagement.

	Estimate	S.E.	Est./S.E.	Two-tailed p-value
Total effect	0.279	0.112	2.496	0.013
Total indirect	0	0.002	0.173	0.862

Log-likelihood = 10076670.790. AIC = -20153267.581. BIC = -20152855.297. Sampe-size adjusted BIC = -20152972.885. R-square (negative emotion) = 0 (SE = .019, p-value = .985).

Table S6. Total and indirect effect between climate relevance, positive emotion, and log retweet engagement.

	Estimate	S.E.	Est./S.E.	Two-tailed p-value
Total effect	0.283	0.113	2.507	0.012
Total indirect	0.014	0.008	1.609	0.108

Log-likelihood = 9680888.949. AIC = -19361703.898. BIC = -19361291.615. Sample-size adjusted BIC = -19361409.202. R-square (positive emotion) = .011 (SE = .003, p = .001).
